# Increased prevalence of insecticide resistance in *Anopheles coluzzii* populations in the city of Yaoundé, Cameroon and influence on pyrethroid-only treated bed net efficacy

**DOI:** 10.1051/parasite/2021003

**Published:** 2021-02-02

**Authors:** Roland Bamou, Edmond Kopya, Leslie Diane Nkahe, Benjamin D. Menze, Parfait Awono-Ambene, Timoléon Tchuinkam, Flobert Njiokou, Charles S. Wondji, Christophe Antonio-Nkondjio

**Affiliations:** 1 Vector-Borne Diseases Laboratory of the Applied Biology and Ecology Research Unit (VBID-URBEA), Department of Animal Biology, Faculty of Science of the University of Dschang P.O. Box 067 Dschang Cameroon; 2 Laboratoire de Recherche sur le Paludisme, Organisation de Coordination pour la lutte contre les Endémies en Afrique Centrale (OCEAC) B.P. 288 Yaoundé Cameroon; 3 Faculty of Sciences, University of Yaoundé I P.O. Box 337 Yaoundé Cameroon; 4 Vector Biology, Liverpool School of Tropical Medicine, Pembroke Place Liverpool L3 5QA United Kingdom; 5 Centre for Research in Infectious Disease (CRID) P.O. Box 13591 Yaoundé Cameroon

**Keywords:** *An. coluzzii*, Insecticide Resistance, Susceptibility, LLINs, Experimental-hut, Bioassay, Cameroon

## Abstract

In Cameroon, pyrethroid-only long-lasting insecticidal nets (LLINs) are still largely used for malaria control. The present study assessed the efficacy of such LLINs against a multiple-resistant population of the major malaria vector, *Anopheles coluzzii,* in the city of Yaoundé via a cone bioassay and release-recapture experimental hut trial. Susceptibility of field mosquitoes in Yaoundé to pyrethroids, DDT, carbamates and organophosphate insecticides was investigated using World Health Organization (WHO) bioassay tube tests. Mechanisms of insecticide resistance were characterised molecularly. Efficacy of unwashed PermaNet^®^ 2.0 was evaluated against untreated control nets using a resistant colonised strain of *An. coluzzii*. Mortality, exophily and blood feeding inhibition were estimated. Field collected *An. coluzzii* displayed high resistance with mortality rates of 3.5% for propoxur (0.1%), 4.16% for DDT (4%), 26.9% for permethrin (0.75%), 50.8% for deltamethrin (0.05%), and 80% for bendiocarb (0.1%). High frequency of the 1014F west-Africa *kdr* allele was recorded in addition to the overexpression of several detoxification genes, such as *Cyp6P3, Cyp6M2, Cyp9K1, Cyp6P4 Cyp6Z1* and *GSTe2*. A low mortality rate (23.2%) and high blood feeding inhibition rate (65%) were observed when resistant *An. coluzzii* were exposed to unwashed PermaNet^®^ 2.0 net compared to control untreated net (*p* < 0.001). Furthermore, low personal protection (52.4%) was observed with the resistant strain, indicating reduction of efficacy. The study highlights the loss of efficacy of pyrethroid-only nets against mosquitoes exhibiting high insecticide resistance and suggests a switch to new generation bed nets to improve control of malaria vector populations in Yaoundé.

## Abstract (fr)

Au Cameroun, moustiquaires insecticides de longue durée (MILDA) contenant uniquement des pyréthrinoïdes sont encore largement utilisées pour lutter contre le paludisme. La présente étude a évalué l’efficacité de ces MILDA contre une population multi-résistante du principal vecteur du paludisme, *Anopheles coluzzii*, de la ville de Yaoundé, en utilisant un test de bio-efficacité et la technique de lâchage-recapture dans des cases-pièges expérimentales. La sensibilité des moustiques collectés sur le terrain à Yaoundé aux pyréthrinoïdes, au DDT, aux carbamates et aux insecticides organophosphorés a été étudiée à l’aide de tests en tube de bio-essai de l’Organisation Mondiale de la Santé (OMS). Les mécanismes de résistance aux insecticides ont été caractérisés au niveau moléculaire. L’efficacité du PermaNet^®^ 2.0 non lavé a été évaluée par rapport à une moustiquaire contrôle non traitée en utilisant une souche colonisée résistante d’*An. coluzzii*. La mortalité, le taux d'inhibition de gorgement ont été estimées. Les *An. coluzzii* collectés sur le terrain ont montré une résistance élevée avec des taux de mortalité de 3,5 % pour le propoxur (0,1 %), 4,16 % pour le DDT (4 %), 26,9 % pour la perméthrine (0,75 %), 50,8 % pour la deltaméthrine (0,05 %) et 80% pour le bendiocarbe (0,1 %). Une fréquence élevée de l’allèle 1014F kdr ouest-africain a été enregistrée en plus de la surexpression de plusieurs gènes de détoxification tels que Cyp6P3, Cyp6M2, Cyp9K1, Cyp6P4 Cyp6Z1 et GSTe2. Un faible taux de mortalité (23,2 %) et un taux élevé d'inhibition de gorgement (65 %) ont été observés lorsque les *An. coluzzii* ont été exposés à une moustiquaire PermaNet^®^ 2.0 non lavée par rapport à une moustiquaire témoin non traitée (*p* < 0,001). De plus, une faible protection individuelle (52,4 %) a été observée avec la souche résistante indiquant une réduction de leur efficacité. L’étude met en évidence la perte d’efficacité des moustiquaires aux pyréthrinoïdes contre les moustiques présentant une résistance élevée aux insecticides et suggère le remplacement de ces moustiquaires par des moustiquaires de nouvelle génération pour améliorer le contrôle des populations de vecteurs du paludisme à Yaoundé.

## Introduction

Malaria is a major cause of morbidity and mortality in Sub-Saharan Africa, and in 2019 the disease was responsible for over 409,000 deaths and 229 million cases [60]. In the absence of a vaccine, disease prevention relies mostly on the use of vector control measures with insecticide-based methods such as long-lasting insecticidal nets (LLINs) and indoor residual spraying (IRS) as the main interventions [13, 37, 56]. According to the World Health Organization (WHO), close to 1.9 billion mosquito nets have been distributed across Africa since 2000 [59]. The massive scale-up of insecticide treated nets (ITNs) and LLINs between 2000 and 2015 across the world is considered to have adverted 630 million malaria cases [13]. However since 2015, increases in malaria incidence and disease transmission have been reported in many parts of the world [60], suggesting a decrease in the efficacy of LLINs and IRS used until now as core interventions [6, 34, 46]. Several factors could be responsible for the poor performance of control measures, including low usage rates of bed nets [31], changes in the biting and resting behaviour of mosquitoes [9, 18, 25, 31], and the rapid expansion of insecticide resistance in vector populations [4, 9, 10, 18, 33, 53]. The rapid expansion of insecticide resistance resulting from the massive use of insecticides in both agriculture and public health, is considered a major threat limiting the performance of current control tools [41, 62]. Several mechanisms such as target site resistance, notably knockdown resistance (*kdr*) [4, 9, 10], modified acetylcholinesterase Ace-1^R^ [10, 19], the overexpression of detoxification genes [10, 19, 47, 53, 61], or cuticular resistance [7, 8, 10, 50, 63] have been reported to induce resistance to insecticides in vector populations. However, despite the diversity of resistance mechanisms and the increasing prevalence of insecticide resistance in vector populations, there is still not enough evidence from the field on how insecticide resistance affects the efficacy of treated nets. Recent studies on *Anopheles funestus* indicated that the expansion of pyrethroid resistance mediated by metabolic mechanisms such as monoxygenase P450 [32, 55] and glutathione s-transferase GST [30] was impacting the efficacy of nets.

In Cameroon, malaria remains a major public health concern in both urban and rural settings [9, 18]. Mosquitoes from the *Anopheles gambiae* complex are the main vectors of malaria across the country [2]. Studies conducted so far have suggested rapid expansion of insecticide resistance in these vector populations and in *An. funestus* [4, 5]. Despite the rapid expansion of insecticide resistance in vector populations, and the availability of new generation nets that are more efficient against insecticide resistant mosquitoes species, pyrethroid-only bed nets are still largely used by the population to minimize the malaria burden [43]. Yet there are still not enough data on the efficacy of pyrethroid-only treated nets against insecticide-resistant *Anopheles gambiae s.l.* populations in Cameroon, particularly in the city of Yaoundé [3–5, 15, 30]. Experimental hut trials are commonly used to assess the efficacy of insecticide-treated bed nets [12, 34, 38, 58]. Many bed net efficacy studies in experimental hut trials have been conducted using free-entering mosquitoes [6, 12, 27, 28, 30, 38, 54]. Others used release-recapture mosquitoes [17, 45]. The present study was conducted to assess the efficacy of pyrethroid-only LLINs against a multi-resistant *Anopheles coluzzii* population in the city of Yaoundé using both an experimental hut trial and cone bioassay.

## Materials and methods

### Ethics approval and consent to participate

The study was conducted under ethics clearance No. 2016/01/685/CE/CNERSH/SP issued by the Cameroon National Ethics (CNE) Committee for Research on Human Health.

#### Mosquito colonies

Mosquitoes used for the study included two susceptible laboratory colonies (*An. coluzzii* Ngousso and *An. gambiae* Kisumu), a resistant colony (*An. coluzzii*) maintained at the OCEAC (Organisation de Coordination pour la lutte Contre les Endémies en Afrique Centrale) Malaria Research insectary, and wild *An. coluzzii* reared from larvae collected in Yaoundé (3°520 N; 11°310 E). The field population of *An. coluzzii* was constituted after collecting mosquitoes (in December 2017) in different habitats, including temporary water collections, puddles and semi-permanent sites in order to capture the genetic variability of the species in Yaoundé. The different groups of mosquitoes (susceptible strains, resistant colony, and field collected *An. coluzzii*) were used for insecticide bioassays involving the WHO tube test and cone test.

#### Establishment of a resistant laboratory colony

Anopheline larvae, collected in standing water collections as described above, were pooled and reared in the laboratory at optimal temperature and humidity (28–30 °C and 70–80% relative humidity). Larvae were reared in trays of 500 cm^2^, each tray containing 50–100 larvae and were fed using Tetramin^®^ babyfish food. After emergence, males were separated from females, then each group was exposed to deltamethrin 0.05%, according to WHO guidelines [58]. Mosquitoes surviving 24 h after exposure were pooled for mating and subsequent egg-laying. A resistant colony was established by regular selection of mosquitoes, exposing 3–5 day-old unfed females and males to 0.05% deltamethrin for three generations.

#### Insecticide susceptibility test

Adult female *An. gambiae s.l.* mosquitoes, obtained from field-collected larvae were exposed to insecticide impregnated papers alongside the *An. gambiae* Kisumu and *An. coluzzii* Ngousso susceptible strains and the *An. coluzzii* resistant colony, following WHO guidelines [58]. Mosquitoes were exposed to the following insecticide concentrations: deltamethrin 0.05%, permethrin 0.75%, DDT 4%, bendiocarb 0.1%, propoxur 0.1%, malathion 5% and fenitrothion 1%. In addition, the synergistic effect of piperonyl butoxide (PBO) on the mortality of wild collected *An. gambiae s.l.* was also tested using the WHO tube bioassay to assess possible implication of monoxygenase-based metabolic resistance (P450). The susceptible strains *An. gambiae* Kisumu and *An. coluzzii* Ngousso were used to assess the efficacy of the impregnated papers. *Anopheles gambiae s.l.* females aged 3–5 days, were introduced in batches of 20–25 mosquitoes per tube and exposed to insecticide-impregnated papers. After 1 h exposure, mosquitoes were transferred to observation tubes and then fed with a 10% sugar solution. The number of dead mosquitoes was recorded after 24 h post-exposure. During each bioassay, a batch of mosquitoes used as a control were exposed to untreated papers. For tests with PBO, batches of 20–25 individuals were first placed in a tube with PBO paper for 1 h before being exposed to deltamethrin 0.05% or permethrin 0.75%. For each insecticide, four replicates and two controls (untreated paper) were run. Bioassays on susceptible laboratory-reared mosquitoes, *An. gambiae* Kisumu and *An, coluzzii* Ngousso strains were run before bioassays on wild collected *An. gambiae s.l.* All the impregnated papers used for the study were obtained from the Universiti Sains Malaysia and kept at 4 °C before and after each test.

#### WHO cone test

Mosquitoes were also exposed using WHO cone bioassays to check the efficacy of PermaNet^®^ 2.0 treated nets. The susceptible strains (Kisumu and Ngousso), and the selected colony of resistant *An. coluzzii* females were used. A number of mosquitoes varying from 5 to 10 aged 3–5 days were released in a cone fitted with a treated net (PermaNet^®^ 2.0) and exposed for 3 min according to WHO guidelines [58]. Similar bioassays were conducted with untreated nets used as a control. Tests were conducted with the ceiling and the sides of the nets. After exposure, mosquitoes were transferred to cups and allowed to feed on cotton wool soaked with a 10% sugar solution then left for observation for 24 h. At the end of this period, the number of dead and surviving mosquitoes was recorded.

#### *Anopheles gambiae s.l.* identification and characterisation of insecticide resistance

A sample of mosquitoes from the resistant strain was used for species identification and *kdr* mutation detection, while field collected *Anopheles spp.* were used for both target site mutation and metabolic-based insecticide resistance gene detection (gene expression analysis). For *An. gambiae s.l.* identification, the protocol described by Santolamazza *et al.* [49] was used. Target site mutations (L1014F, L1014S and G119S) were assessed using the protocol described by Bass *et al.* [11], while gene expression analysis was performed using newly developed quantitative reverse transcription-real-time PCR (qRT-PCR) 3-plex assays, as described in Bamou *et al.* [10]. Genes analysed included *Cyp6m2, Cyp6p3, Cyp6p4, Cyp6p1, Cyp9k1, Cyp4g16* and *GSTE2*. For normalisation purposes, the RPS7 gene was used [29].

#### Bed nets used

The treated bed nets used for cone bioassays and the experimental hut trial were PermaNet^®^ 2.0 bought from the market. The nets are 100% polyester coated with 1.8 g/kg of deltamethrin. The mosquito nets were intentionally holed according to World Health Organization Pesticide Evaluation Scheme (WHOPES) directives [36, 57] in the middle part, on a surface representing approximately 0.8% of the total surface. This was to mimic the real situation of LLINs in villages where LLINs have holes due to frequent use and to see whether the net was just a physical barrier and whether the insecticide was able to prevent blood feeding, even if the net had holes. Six holes of 4 cm^2^ (2 cm × 2 cm) were made in each net (unwashed PermaNet^®^ 2.0 and untreated nets), 2 holes in each of the long-side panels, and one hole at each end (i.e. head and foot-side panels).

#### Assessment of hut lethality

Prior to the experimental hut trial, a series of bioassays were conducted to assess the lethal effect of huts using susceptible mosquitoes (Kisumu and Ngousso). Adult mosquitoes were exposed to various surfaces in the huts including the ceiling, floor, doors, walls and screening-mesh of the veranda. Bioassays were performed using WHO testing cones attached to surfaces with masking tape. Ten 3–5-day-old females of the *An. gambiae* Kisumu and *An coluzzii* (Ngousso) strains were put into each cone for 30 min [1]. After exposure, they were removed from the cones and put into plastic cups covered with untreated mosquito net and given access to 10% glucose solution, and mortality was recorded after 24 h.

#### Release-recapture experimental hut trials

Five West African style experimental huts with a veranda, where mosquitos are allowed to enter freely, were used for the study, with slight modifications. The station is located in Mibelon and further details can be found in Menze *et al.* [29]. Window slits were closed, preventing wild mosquitoes from entering and released mosquitoes from escaping [17, 45]. The protocol used was approved by the National Ethics Committee (No. 2016/01/685/CE/CNERSH/SP), and each volunteer participating in the study signed the informed consent form after explanation of the objective of the study and risks associated with their participation. In addition, volunteers received medical care in case of illness during the entire duration of the study. After recruitment, volunteers were trained before the trials and only five collectors with similar body size and good skills were retained for the study. Every night, four volunteers slept each under a treated net (unwashed PermaNet^®^ 2.0) in a hut, and one volunteer was placed under an untreated net used as a control from 8 pm to 6 am. To prevent volunteers leaving experimental huts during the night, drinking water was provided and a urinal receptacle made available. When volunteers were under their net, a batch of 100 pyrethroid-resistant mosquitoes were introduced into the hut and the catches were made in the morning at 6 am. Mosquitoes were collected individually in tubes and then kept in bags labelled according to the place of capture/collection or compartment of the hut: inside the mosquito net, in the hut (outside the mosquito nets, on the walls and ceiling), and in the veranda-trap. The huts were checked each afternoon before the catches. To create a contrast between the ground and mosquitoes, a white plastic tissue was placed on the ground and replaced with a clean one each day before the experiment. During each mosquito release session, the sleepers carried out circular permutation between the huts, in order to avoid bias due to the effect of preferential attraction that they could offer. The status of mosquitoes collected each morning was registered (unfed, blood-fed, dead, alive) as reported elsewhere [48], and those alive were put in small paper cups with access to 10% glucose solution, with 24 h observations and recording for any cases of possible delayed mortality. The mosquitoes collected were morphologically identified using a binocular microscope [21, 22], to verify the intrusion of other species into the huts during the day when the huts were cleaned.

#### Outcomes measures

The following outcomes were measured to assess the efficacy of the unwashed PermaNet^®^ 2.0 nets in experimental huts:

- *Exophily (Excito-repellency)*: the proportion of mosquitoes found exited in the veranda trap. Exophily (%) = 100 × (*Ev*/*Et*) where *Ev* is the total number of mosquitoes found in the veranda and *Et* is the total number of both inside the hut and veranda.
- *Blood feeding rate (BFR)*: this rate was calculated as follows: Blood feeding rate = (*N* mosquitoes fed) × 100/total *N* mosquitoes. Where “*N* mosquitoes fed” was the number of mosquitoes fed, and “total *N* mosquitoes” was the total number of mosquitoes collected.
- *Blood feeding inhibition (BFI)*: the reduction in blood feeding in comparison with the control hut. Blood feeding inhibition is an indicator of personal protection (PP). More precisely, the personal protection effect of each bed net is the reduction of blood feeding percentage induced by the net when compared to the control. The protective effect of each bed net can be calculated as follows: Personal protection (%) = 100 × (*Bu* – *Bt*)/*Bu*, where Bu is the total number of blood-fed mosquitoes in the huts with untreated nets, and *Bt* is the total number of blood-fed mosquitoes in the huts with treated nets [57].
- *Immediate and delayed mortality*: the proportion of mosquitoes entering the hut that are found dead in the morning (immediate mortality) or after being caught alive and held for 24 h with access to sugar solution (delay mortality). In this study, we focused on the overall mortality calculated as follows: Mortality (%) = 100 × (*Mt*/*MT*) where *Mt* is the total number of mosquitoes found dead in the hut, and *MT* is the total number of mosquitoes collected in the hut [57].

#### Statistical analysis

Differences in proportional outcome variables (mortality, blood feeding inhibition, and exophily) between treatment (unwashed PermaNet^®^ 2.0) and control (untreated net) were analysed using logistic regression, after adjusting for the effect of the sleeper. The number of mosquitoes entering the nets, the numbers succeeding in feeding, and the numbers killed were analysed using negative binomial regression with adjustments for sleepers. Insecticide resistance results from bioassays were interpreted following WHO guidelines [58]. Chi square tests were used to compare mortality between mosquitoes exposed to pyrethroids and the pyrethroids-PBO combination. Calculation of fold-change, 95% confidence intervals, and statistical significance were performed using the Pfaffl method [40]. These analyses were done on STATA version 11.

## Results

### *Insecticide susceptibility status of* An. gambiae *populations*

Field *An. gambiae s.l.* females were found to be resistant to DDT, pyrethroids and carbamates and susceptible to organophosphates (Table 1). The mortality rate was 50.8% for deltamethrin, 26.9% for permethrin, 4.16% for DDT, 3.33% for propoxur, and 85% for bendiocarb (Table 1). Female *An. gambiae s.l.* pre-exposed to PBO showed an increase in susceptibility to both permethrin (with the mortality rate increasing from 26.9% to 35%) (*p* < 0.0001) and deltamethrin (with the mortality rate increasing from 50.8% to 72%) (*p* < 0.0001). The resistant *An. coluzzii* population exhibited a mortality rate of 35% (*n* = 100; 95% CI: 24.37 – 48.67) and 8% (*n* = 100; 95% CI: 3.45 – 15.46) for deltamethrin and permethrin, respectively (Table 1).

**Table 1:** Mortality rate of *Anopheles gambiae* s.l. from Yaoundé exposed to certain insecticides.

	*An. coluzzii* resistant strain		Field *An. gambiae* s.l.		*An. coluzzii Ngousso strain*		*An. gambiae Kisumu strain*	
	*N*	%Mortality [95% CI]	*N*	%Mortality [95% CI]	*N*	%Mortality [95% CI]	*N*	%Mortality [95% CI]
Permethrin	100	8 [3.5 – 15.8]	156	26.9 [19.4 – 36.4]	100	100 [81.4 – 121.6]	100	100 [81.4 – 121.6]
Deltamethrin	100	35 [24.4 – 48.7]	258	50.8 [42.5 – 60.3]	100	100 [81.4 – 121.6]	100	100 [81.4 – 121.6]
DDT	–	–	120	4.16 [1.4 – 9.7]	100	98 [79.6 – 119.4]	100	99 [80.5 – 120]
Propoxur	–	–	120	3.33 [0.9 – 8.5]	100	100 [81.4 – 121.6]	100	100 [81.4 – 121.6]
Bendiocarb		–	100	86 [68.8 – 106.2]	100	100 [81.4 – 121.6]	100	100 [81.4 – 121.6]
Malathion	–	–	120	100 [82.9 – 119.6]	100	100 [81.4 – 121.6]	100	100 [81.4 – 121.6]
Fenitrothion	–	–	120	100 [82.9 – 119.6]		–		–
PBO Deltamethrin	–	–	120	71.66 [57.3 – 88.5]		–		–
PBO Permethrin	–	–	100	35.0 [24.4 – 48.7]		–		–

*N*: number of mosquitoes tested; 95% CI: 95% confidence interval.

### Molecular characterisation of insecticide resistance

A subsample of 100 *An. gambiae s.l.* females from the field and the resistant strain were used for species identification and insecticide resistance characterisation. All mosquitoes screened were *An. coluzzii.* For target site mutation, only the L1014F allele (West Africa Knockdown resistance allele), in both the homozygote (*n* = 98) and heterozygote state (*n* = 2), was detected. The *kdr* allele frequency in the resistant colony was 99%.

The expression profiles of eight genes implicated in insecticide resistance were analysed in the field collected mosquitoes from Yaoundé. All genes screened except *Cyp6p1* showed an overexpression profile compared to susceptible mosquitoes (Table 2). The highest overexpression was seen for gene *Cyp6m2*, with a fold change of 4.5. Other highly overexpressed genes also included *Cyp9k1*, Cyp6p3 *Cyp6z1*, and *Cyp4g16*.

**Table 2:** Gene expression analysis of field collected *Anopheles coluzzii* from Yaoundé compared to the susceptible Ngousso (NG) and Kisumu (KIS) strains.

	Fold changes (95% CI)							
	*CYP6P3*	*CYP6M2*	*CYP9K1*	*CYP6P4*	*CYP6Z1*	*GSTE2*	*CYP6P1*	*CYP4G16*
vs. KIS	**4.22**	**4.50** *	**7.30** *	**3.80** *	**3.40** *	**2.55**	0.85	**1.75**
	(2.63–6.96)	(2.93–6.10)	(4.03–19.5)	(2.56–5.09)	(2.36–4.93)	(1.99–3.12)	(0.71–0.97)	(1.20–3.76)
vs. NG	1.18	**2.75** *	**2.8** *	**2.99** *	**2.52** *	0.71	0.45	1.21
	(0.80–1.78)	(1.73–3.75)	(1.76–5.71)	(1.93–4.17)	(1.64–3.42)	(0.56–0.87)	(0.36–0.56)	(0.92–1.55)

Bold letters indicate statistically significant upregulation.

* Asterisks indicate consistent upregulation compared to both susceptible strains (*p* < 0.05).

### Cone bioassays with PermaNet^®^ 2.0 nets

Efficacy of the deltamethrin-coated net (unwashed PermaNet^®^ 2.0) was also evaluated using the resistant colony of *An. coluzzii*. For these tests, 269 females were exposed to PermaNet^®^ 2.0 nets, while 40 females were exposed to untreated nets (control). An average mortality rate of 44.24% was recorded after exposing mosquitoes to PermaNet^®^ 2.0 nets. For the controls, a mortality rate of 5% (*n* = 40; CI: 0.24 – 7.22) after 24 h observation was recorded (Table 3).

**Table 3:** Knockdown and mortality of different *Anopheles gambiae* s.l. colonies used for cone bioassays.

	Control		PermaNet^®^ 2.0	
	*N*	%	*N*	%
***An. coluzzii* Ngousso strain**	**60**		**60**	
Mortality after 24 h observation	60	0	60	100
Knockdown at 60 min (%)	60	0	60	100
***An. gambiae* Kisumu strain**	**60**		**60**	
Mortality after 24 h observation	60	0	60	100
Knockdown at 60 min (%)	60	0	60	100
***An. coluzzii* Resistant strain**	**40**		**269**	
Mortality after 24 h observation	2	5	119	44.24
Knockdown at 60 min (%)	1	2.5	175	65.05

*N*: number of mosquitoes tested (in bold) or dead (without bold).

### Assessment of experimental hut lethality

Baseline analysis using WHO cone bioassays was conducted on various areas of the hut, including doors, walls, veranda screening-mesh, ceiling, and floor. About 10 mosquitoes were exposed per hut area (50 mosquitoes per hut). The analysis revealed that hut usage in this study had no lethality on the susceptible Kisumu and Ngousso strains. Very low mortality was observed in all huts, with mortality varying from 0% to 4%, and with average mortality of < 2% per hut.

### Experimental hut trials

A total of 4900 resistant *An. coluzzii* females were released in test huts over 8 consecutive days; 3666 were recaptured, representing a recapture rate of 74.8%.

### Exophily

Relative to the untreated control net, the proportion of mosquitoes exiting to verandas was significantly higher in huts with unwashed PermaNet^®^ 2.0 nets (*p* < 0.0001). In the control hut, a 45.2% (316/699) exophily rate was recorded, whereas in huts with PermaNet^®^ 2.0 nets an exophily rate of 57.6% was found.

### Blood feeding rate

Blood feeding rate was also significantly higher in the control compared to the test huts (*p* < 0.001). In the control hut, 14% of females (98) were blood-fed, while in test huts only 5% of mosquitoes were able to blood feed (149/2967). Unwashed PermaNet^®^ 2.0 nets inhibited blood feeding relative to the control at a frequency of 64.18% (Table 4).

**Table 4:** Evaluation of behavioural response of resistant *Anopheles gambiae* s.l. population exposed to PermaNet^®^ 2.0 nets.

	Control	PermaNet^®^ 2.0	*p*-value
Number of released females	900	4000	
Number of recaptured females	699	2967	
Recapture rate (%)	77.67	74.17	
Number collected in room	291	1124	
Number collected in veranda	316	1709	
Collected in net (%)	92 (13.2)	134 (4.5)	
Exophily (%) [95% CI]	45.2 [41.52 – 48.90]	57.6 [55.82 – 59.38]	<0.0001
Induced exophily (%)		22.62	
Number Blood-fed	98	149	
Blood feeding rate (%) [95% CI]	14 [11.45 – 16.59]	5 [4.24 – 5.81]	<0.0001
Blood feeding inhibition (%)		64.18	
Mortality of blood fed mosquitoes (%)	8 (8.2)	40 (26.8)	<0.0001
Number dead	116	687	
Overall mortality rate (%) [95% CI]	16.6 [13.84 – 19.35]	23.2 [21.64 – 24.67]	<0.0001
Corrected mortality		7.82	

### Mortality rate

Immediate mortality varied significantly from 6.3% for control huts to 14.2% for test huts. Immediate mortality induced by PermaNet^®^ 2.0 nets on the resistant *An. coluzzii* population was 7.84%. A total of 116 *An. coluzzii* were found dead after 24 h in the control hut, corresponding to a mortality rate of 16.6%. In the test huts, a delayed mortality rate of 23.2% (655/2967) was observed. The mortality in the test huts was significantly different from that of the control huts (*p* < 0.001). Of the 116 dead *An. coluzzii* collected from control huts, 8 (8.2%) were blood-fed, while in test huts with PermaNet^®^ 2.0 nets, of the 687 dead mosquitoes collected, 40 (1.3%) were blood-fed. The delayed mortality induced by the PermaNet^®^ nets was 8.86% (Table 4).

### Chemical barrier

In the control hut, 92 mosquitoes (13.2%) were collected inside the mosquito net. In the test huts with PermaNet^®^ 2.0 nets, 134/2967 mosquitoes (4.5%) were collected inside the nets. PermaNet^®^ 2.0 nets were found to significantly decrease the rate of mosquitoes entering into the impregnated mosquito nets (*p* < 0.001).

### Personal and community protection

PermaNet^®^ 2.0 nets were found to induce a personal protection rate of 52.04% and a community protection rate of 81.69%, as shown in Table 5.

**Table 5:** Personal and community protection confered by PermaNet^®^ 2.0 nets when exposed to a resistant *Anopheles coluzzii* population.

	Control (*u*)	PermaNet 2.0 (*t*)
Caught mosquitoes (*E*)	699	2967
Blood-fed (*B*)	98	149
**Personal protection** * **(%)**		**52.04**
Overall mortality (*D*)	116	687
**Mass protection** ** **(%)**		**81.69**

* 100 × (*Bu* − *Bt*)/*Bu*;

** 100 × (*Dt* − *Du*)/*Eu* where “*Bu*” is the total number of blood-fed mosquitoes in the huts with untreated nets, and “*Bt*” is the total number of blood-fed mosquitoes in the huts with treated nets. “*Dt*” is the number of dead mosquitoes in huts with treated nets, and *“Du*” the number of dead mosquitoes in the control hut.

## Discussion

Several new-generation nets have been prequalified by the WHO for use in malaria control and elimination. However, the WHO still recommends the use of prequalified pyrethroid-only LLINs as a core intervention in all malaria-endemic settings [60]. Pyrethroid-PBO nets prequalified by WHO are conditionally recommended for deployment where the principal malaria vector exhibits pyrethroid resistance conferred at least in part by a monooxygenase-based metabolic resistance mechanism [60]. In Cameroon, despite the rapid expansion of insecticide resistance in vector populations, pyrethroid-only LLINs are still largely used by the population for malaria prevention because they are the only ones distributed during mass distribution campaigns [43]. The present study was conducted to assess the efficacy of PermaNet^®^ 2.0 nets against a multiple insecticide-resistant *An. coluzzii* strain originating from the city of Yaoundé. Mosquito populations here were reported to express high resistance to DDT, pyrethroids, and carbamates, with resistance mechanisms including the *kdr* allele (West and East *kdr*), and overexpression of detoxification genes such as *Cyp6m2, Cyp6p3, Cyp6z2 Cyp9k1*, *Cyp6z1*, and *Cyp4g16* [3–5, 10]. Efficacy tests conducted with unwashed PermaNet^®^ 2.0 nets suggested low personal protection provided by these nets against resistant *An. coluzzii* populations in the city of Yaoundé. The mortality rate induced by PermaNet^®^ 2.0 nets during the present study was far lower compared to previous studies [27]. The low mortality rate recorded during this study could be consistent with the high frequency of *kdr* alleles and metabolic mechanisms expressed by mosquito populations in the city of Yaoundé [4, 5, 10]. In Benin, the mortality induced by pyrethroid-only bed nets was found to be 30% in an area with high pyrethroid resistant *An. gambiae* populations, whereas it was about 98% in areas with susceptible populations [34]. Nonetheless, continued efficacy of pyrethroid-only treated nets in areas with moderate resistance levels have been reported in many parts of the African continent [15, 17, 20, 26, 27]. Studies conducted so far across Africa, indicated high efficiency of new classes of nets combining the synergist PBO with pyrethroid against resistant mosquito populations compared to pyrethroid-only nets. In Cote d’Ivoire, higher mortality rates were recorded when resistant *An. gambiae* populations were exposed to alpha-cypermethrin + PBO mixture LLINs compared to standard LLINs [38]. In Burkina Faso, high efficiency against resistant *An. gambiae* populations was recorded with PBO-based nets (DawaPlus 3.0 and DawaPlus 4.0) [12]. A recent hut trial conducted in Cameroon using both PermaNet^®^ 2.0, Olyset Plus and PermaNet^®^ 3.0 nets indicated that PBO-based nets induced higher mortality against pyrethroid-resistant *An. funestus* than pyrethroid-only nets [30]. PBO is a synergist that inhibits insecticide resistance induced by the presence of cytochrome P450 metabolic enzymes [14]. The inhibition of P450 enzymes by PBO results in increased efficacy of pyrethroids on resistant mosquito populations [51]. However, it is still difficult to conclude that the increased mortality registered between PBO-based nets and standard LLINs derives from PBO because the original concentration of pyrethroid or the bleed rate of pyrethroid in the pyrethroid-PBO net was found to differ significantly from that in the pyrethroid-only LLINs [26, 39].

A high blood feeding inhibition (BFI) rate of over 64% was recorded and attributed to the irritant effect of pyrethroids [16]. In fact, several pyrethroids such as bifenthrin and deltamethrin are known to induce high irritancy effects on mosquito populations [15, 23, 24]. Recent studies in Cameroon with PermaNet^®^ 3.0 and Olyset Plus [30] also recorded a high irritancy effect on *An. funestus*. In Benin, Olyset and Olyset Plus nets were found to induce a high BFI rate in *An. gambiae* [39]. The high blood feeding inhibition effect of PermaNet^®^ 2.0 nets suggests a possible influence of this tool against resistant mosquito populations.

A high exophilic rate (57.6%) was recorded when resistant mosquitoes were exposed to PermaNet^®^ 2.0 nets. In Cote d’Ivoire and Benin, resistant mosquitoes were instead reported to exhibit a low exophily rate to permethrin, deltamethrin and bifenthrin [26, 34]. This contrasting pattern could be associated with differences in behaviour, resistance intensity, and resistance mechanisms. Importantly, *An. gambiae s.l.* populations from the city of Yaoundé were reported to be highly exophagic [18]. Despite a high LLIN ownership rate, it is estimated that less than 60% of the human population used bed nets properly [42]; in fact, many people stay outdoors late into the night [52]. This situation could have selected exophagic and exophilic behaviour in vector populations. Despite this, a high number of resistant mosquitoes were collected blood-fed in treated nets, confirming their ability to also take blood meals from people sleeping under treated nets. Only a quarter of blood-fed mosquitoes were found dead after a blood meal.

As reported in previous studies, the efficiency of treated nets could depend on the intensity of insecticide resistance and the diversity of detoxification resistance mechanisms. Because resistance characteristics could vary significantly from one epidemiological setting to another, comparison trials of different brands of treated bed nets should be conducted in the same locality, in order to identify nets that are particularly effective against local vector populations.

Although assessment of the washing effect on the net efficacy was not investigated in this study, previous hut trials conducted in Benin and Cote d’Ivoire indicated a significant decrease in PBO content and PBO-based net efficacy (mortality and blood feeding inhibition) after several washes, with PBO nets performing no better than pyrethroid–only LLINs [26, 35]. Despite the decrease in PBO content on the net under natural conditions, the use of PBO-based nets was reported to induce a 33% reduction in malaria incidence in children compared to pyrethroid-only nets after 21 months [44]. Moreover, although the rapid expansion of insecticide resistance could affect pyrethroid-treated net efficacy, it is still not clear whether this is inducing sub-performance of the intervention in the city of Yaoundé. Our study highlights the need for large-scale field effectiveness studies to assess the effect of insecticide resistance on the performance of pyrethroid-treated nets in Yaoundé.

## Conclusion

This study provided evidence of the limited efficacy of the standard treated bed net (PermaNet^®^ 2.0) against pyrethroid-resistant *An. coluzzii* populations in the city of Yaoundé. As previously highlighted by numerous studies, the rapid expansion of insecticide resistance could seriously affect the efficiency of vector control measures implemented in the field. This situation requires the implementation of new control strategies such as the replacement of standard LLINs by PBO-based LLINs or new-generation LLINs, or the addition of new control measures such as IRS or larviciding to preserve existing tools and to manage insecticide resistance. It is urgent that new control tools, such as PBO-based nets, be included in the arsenal of tools used for malaria vector control in Cameroon. However, as done elsewhere, the adoption of this tool will require large-scale randomised controlled trials in order to assess the impact of the intervention on both entomological and epidemiological indicators, as recommended by the WHO [59].

## Abbreviations

LLINs: Long-Lasting Insecticidal Nets; IRS: Indoor Residual Spraying; WHO: World Health Organization; PBO: piperonyl butoxide

## Competing interests

The authors declare that they have no competing interests.

## Funding

This work received financial support from WHO TDR grant 2016/602099-0 and a Wellcome Trust Senior Fellowship in Public Health and Tropical Medicine (202687/Z/16/Z) to CAN. The funding bodies played no role in the design of the study, collection of data, analysis and interpretation of data, nor in writing of the manuscript.

## Authors’ contributions

RB and CAN: conceptualised and designed the study; RB, EK, LDN, BDM, PAA, and CAN performed the field and laboratory experiments. RB performed the statistical analysis. RB, EK, LDN, BDM, PAA, TT, FN, CSW, and CAN critically reviewed and amended the manuscript. RB and CAN wrote the manuscript with input from all authors. All authors read and approved the final manuscript.
